# Supplementary material for: Surface properties and bioactivity of TiO2 nanotube array prepared by two-step anodic oxidation for biomedical applications
Source: R Soc Open Sci. 2019 Apr 24;6(4):181948. doi: 10.1098/rsos.181948 (PMC6502370; doi:10.1098/rsos.181948)
Supplement: Supplementary Figures [file rsos181948supp1.doc]

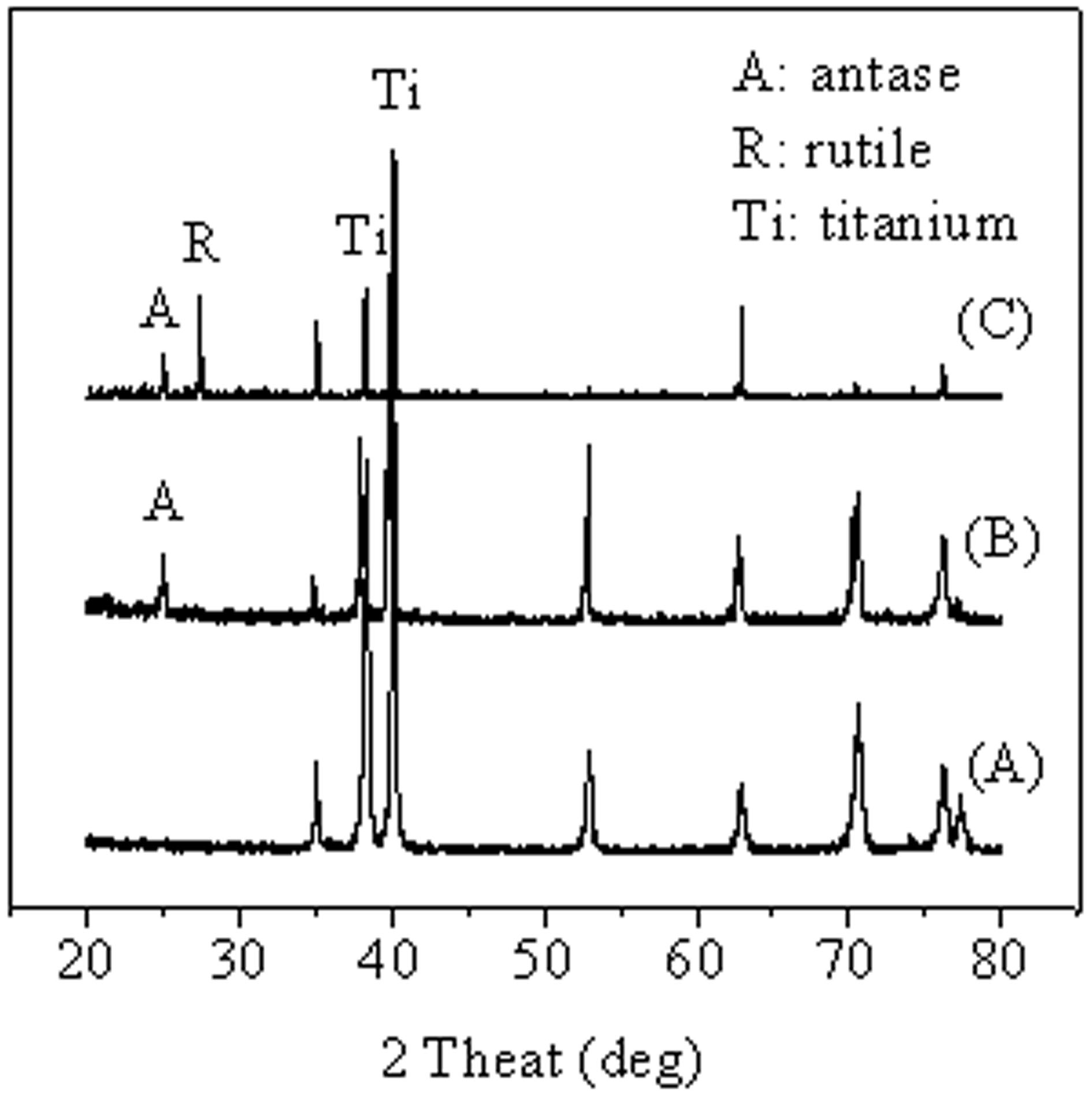


Fig.1 XRD spectrum of nanotube arrays. (A) as-prepared, (B) and (C) after heat treatment at 500℃ and 800℃ respectively

**
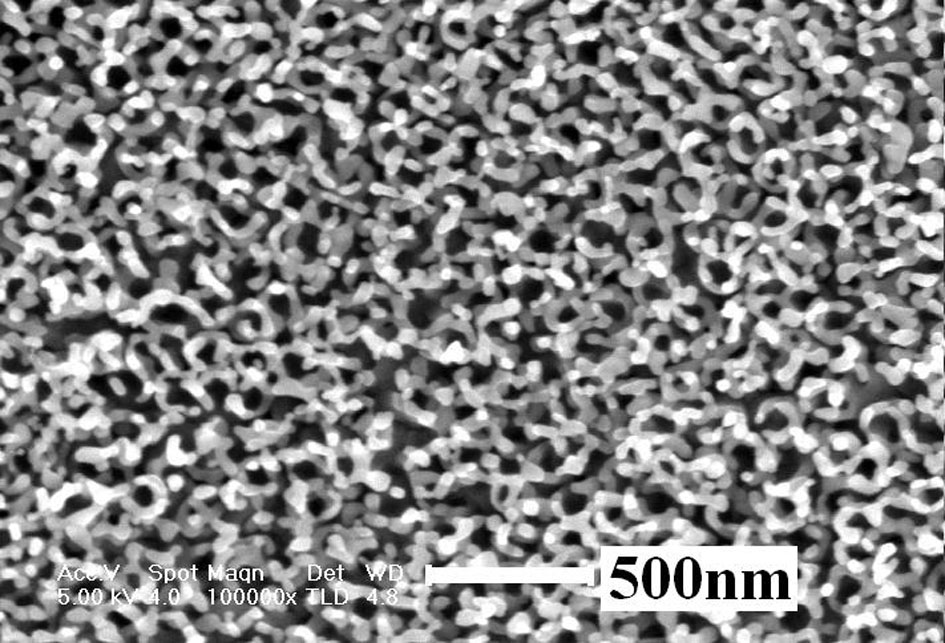
**

Fig.2 FESEM image of the annealed nanotube arrays at 800℃

**
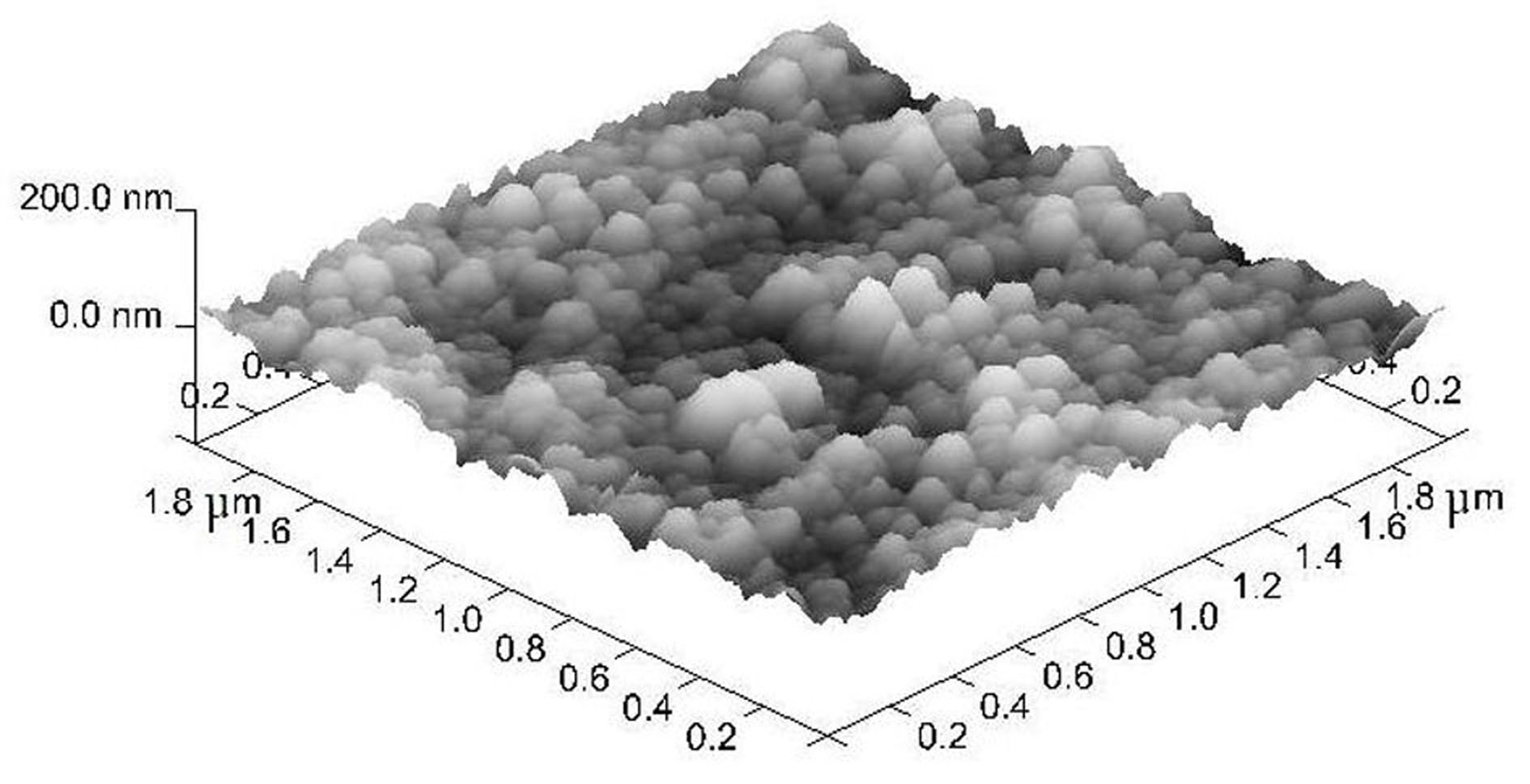
**

Fig.3 AFM image of anodized titanium with nanotube structure.

**
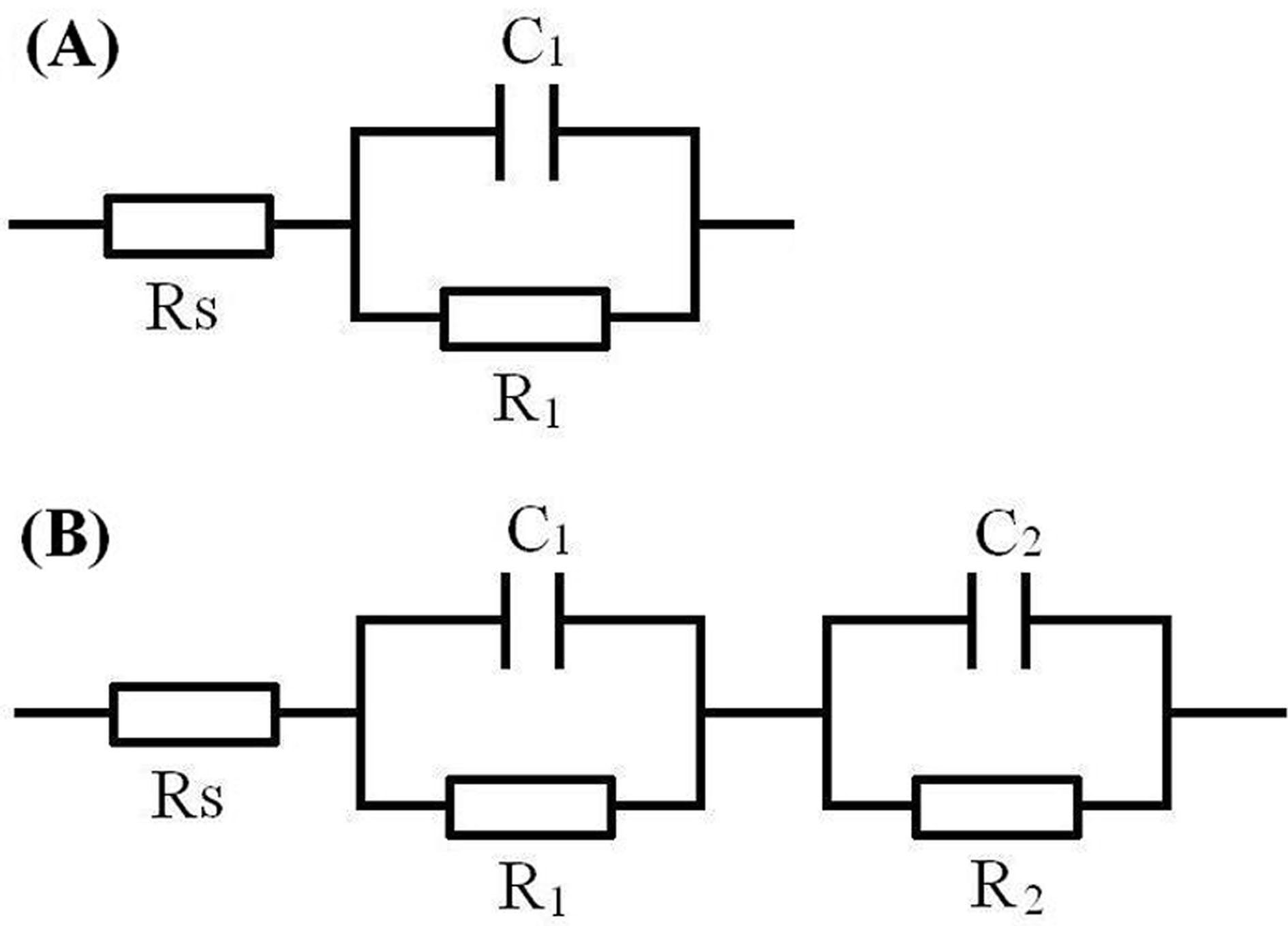
**

Fig.4 Equivalent circuit used for titanium under different oxidation conditions (A) for bare surface and oxide voltage 10V; (B) for oxide voltage 20V and 30V.

**
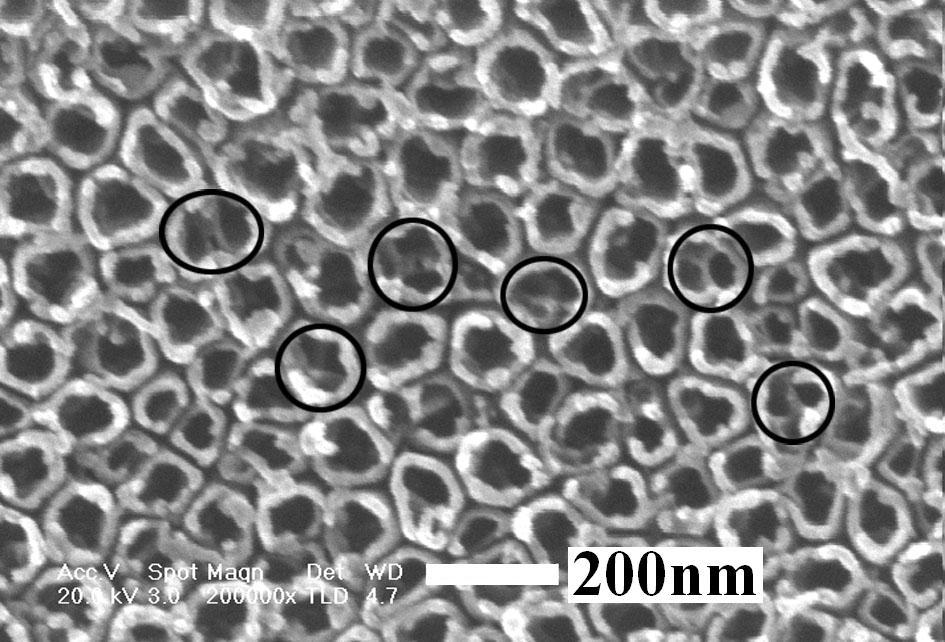
**

Fig.5 SEM images of the nanostructure formed on pure titanium in 0.6vol%HF at 20V for 25min.
